# A personal reflection upon navigating into a senior academic role

**DOI:** 10.3389/fsoc.2023.979691

**Published:** 2023-06-21

**Authors:** Bernadine D. Idowu

**Affiliations:** ^1^School of Biomedical Sciences, University of West London, London, United Kingdom; ^2^Centre for Oral, Clinical and Translational Science, King's College London, London, United Kingdom

**Keywords:** BME, academics, inequalities, HE, representation

## Abstract

There are 22,795 University professors in the UK, where 6,340 are women and only 40 are Black women, whilst Asian women are a few more in number. This clearly demonstrates the uncommon narrative of the under-representation of Black minority ethnic (BME) academics in higher education (HE) which has been written about in detail. In contrast, it is rare to read reports on initiatives to successfully navigate senior academic posts. In this article, I will describe two initiatives that I have developed and organized to successfully navigate senior BME academic posts, which have impacted my journey. The first initiative was to understand why postdoctoral researchers were “post-docing” for years, having not been successful in making the transition to lecturers. What was hindering the transition? I was one of them, and some of my female peers as well, who incidentally left HE. I was determined not to leave. I again thought about how to tackle it. It is a known fact that hearing successful BME people experiences and journeys and also understanding how they navigated HE can be powerful. In addition, empowering oneself with additional skills including mentoring, networking, applying for positions, not excluding ourselves due to the lack of confidence, and finally, the importance of having a work–life balance is important as health is wealth. I used this to put together the BME Early Career Researcher (ECR) conference—How to Stay in Academia. After 6 years, it is still going strong. In this article, I will share the impact made over the years which will include testimonies and promotions, including my recent promotion to an associate professor. The second initiative was to understand the barriers and challenges of senior lecturers being promoted to readers and professors. Having successfully transitioned to a lecturer, being overlooked for promotion was now an issue. The project was conducted in 2016/17 at KCL as part of the action plans that needed to be delivered having been a recipient of the Bronze Race Equality Charter Mark. I was provided with a cohort of 51 names of BME staff from different disciplines and was directed to see how I would engage them to hear their experiences. My first concern was that the staff would have engaged in previous initiatives with little or no benefits to them; however, this did not deter me. I thought of the best approach which commenced with a phone interview and then followed up with a focus group, ending with an informal conversation with the Principal of the University. Within 6 months, a BME male was promoted to professor. After a year, both genders were promoted to associate professors (readers) and professors, and to date, I am aware of at least 10 promotions. In both examples, I will demonstrate the support from our allies, some of whom are senior leaders that have openly supported us in our journey. This article will demonstrate a slight shift in the narrative, but a lot more needs to be done, and I am convinced the time is right to start pushing for more. This special issue is an example.

## Introduction

Black women are considered as a rarity in academia (Opara, 1997, 2020; Wilson, 1997; Owusu-Kwarteng, 2021). In 2021, advanced HE reported that there are 22,795 university professors in the UK, where 6,340 are women and only 40 are Black women (Advance HE, 2021). These data can certainly be perceived as racial inequality in higher education. There is a plethora of reports which demonstrate the lack of diverse appointments as professors or working in other senior roles. This has been significantly and continuously lower than white staff across UK universities (Mirza, 2009; Grove, 2015; McGregor, 2017; Adams, 2018; Coughlan, 2018; Mitchell and Saunders, 2019; the situation for women was highlighted in Women in Higher Education Network in 2022). This is impacting students' feedback as reported by Francis (2022), “*the lack of senior black academics had a direct impact on students' experiences and education*.” She continues “*it means a lack of our voices, knowledge, works, and histories in the curriculum itself*.” Morgan (2016) also comments on this in his article ‘Why is my professor still not black?' (Morgan, 2019).

Listening to the experiences of ECR encourages me to see how I can change the narrative and increase the numbers of BME ECR in academia. I am keen to reflect on how I can encourage staff to seek promotion in both initiatives discussed in this article. I have a career coach who has supported me in my goals. They once said to me that I was a do-er which is true, as I identify an issue, and using my network, I set about addressing it. I strongly believe for the future BME generations who aspire to become academics, which will increase the pool, they need to see people that look like them teaching them, as mentioned by some of mine and other BME undergraduate and graduate students (Morgan, 2014; Havergal, 2016; Idowu-Onibokun, 2017, 2021; Morgan, 2020b). Professor Winston Morgan exemplifies the impact BME lecturers can have in these spaces, “*Having black professors designing/informing the curriculum will reduce the Eurocentric content of the curriculum and make it more accessible, engaging, and inspirational*” (Idowu-Onibokun, 2018).

The data for OfS (Office for Students, 2020) tells us year on year that the numbers of BME students attending universities are increasing. However, despite this, it is being reported that BME students after graduating are less likely to progress to go on to study a research degree as some do not have a 2:1 or first-class degree (Grove, 2015); hence, the pipeline is broken. It is important that universities, where possible, put initiatives in place to make all students feel they are being supported. A quick win would be a peer mentoring project (Idowu, 2022).

Some, however, do progress on the successful completion of their PhD, to do a postdoctoral programme, they are usually referred to as early career researchers (ECRs). However, after completing a couple of postdoctoral projects, many are unable to transition to a permanent academic position, namely a lectureship, and as a consequence leave academia. The fixed-term contracts give no job security (Grove, 2015; Morgan, 2020a), and again the pipeline is broken; hence, the number of staff moving onto the professional level remains low.

King's College London (KCL) was one of eight institutions that awarded the Race Equality Charter Mark in 2014 (Advance HE, 2020). By being awarded the bronze mark, it demonstrates that the institution has committed to equality and inclusion and its ability to deliver on our action plan. The data obtained from KCL identified that BME staff are under-represented at the lecturer level and above. Given the under-representation of BME academics at more senior levels, as part of the university action plan, Kings committed to improve representation from the lecturer to the professorial level, which includes the following academic ranks: lecturer, senior lecturer, reader (also known as an associate professor), and, finally, professor. Representation is so important as both students and staff alike, who aspire to be in these positions, would be empowered and are more likely to reach out to the academics to be mentored.

This article will therefore seek to provide a personal reflection into navigating a senior academic role using two initiatives which are success stories, first to increase the numbers of Black lecturers through a very successful yearly conference now in its 7th year and the other, engaging senior lecturers to actively put themselves forward for promotion to become professors.

## First initiative—Under-representation and barriers to transition

My first initiative was to tackle the under-representation of postdoctoral researchers making the transition to lecturers as the data obtained from KCL show it is very low. I then explored ways to empower postdoctoral researchers also referred to as early career researchers that wanted a career in academia to feel empowered to navigate the transition.

I addressed this by organizing a conference in order to create a safe space to discuss both the challenges and what steps were needed to be actioned. I decided not to limit it to KCL ECR as under-representation is not unique to King's but to many universities within the UK. As it was not possible to predict the level of interest, I decided to limit the attendees to London universities and refer to it as a pilot study. After evaluating the findings, to then extend the reach further, attendees from outside London were invited to subsequent conferences. However, I initially invited 10 representatives from 10 universities in the UK who were either academics or professional staff supporting postdoctoral research to be part of the first committee organizing the pilot conference.

I conducted research into previous HE conferences addressing these issues so as not to re-invent the wheel but discovered no ECR conferences covering many disciplines that had been delivered, and the nearest to this was staff networks (Hertfordshire), or subject-specific education (UCL); therefore, such a conference was needed as there was a gap for it.

The committee liked the format I suggested, namely a 1-day conference, a morning session with a welcome speech from the founder (myself) and then an opening address from a senior leader of the university, demonstrating support, followed by a keynote speech from an established academic and finally a panelist mixed with ECRs. The afternoon session consisted of empowerment sessions, which were short workshops, covering the importance of mentoring/sponsorship, networking, applying for fellowships, and having a work–life balance. The sessions were run concurrently, enabling everyone to opt out of a session, rather than having to choose from a parallel session. The day ended with a networking event, with refreshments. All delegates were, however, encouraged to network throughout the day.

Approximately a month after the conference, a follow-up session is organized for those that have shown evidence of being pro-active. These delegates are invited to attend a free afternoon workshop organized by the Fistral Training and Consultancy who work with researchers and professional and support staff across Europe. The title of the workshop is “Maximizing your potential” which aims to gain insights into individuals' key personal and professional strengths and motivations.

Now, in its 7th year, the yearly conference has been conducted at various London universities. The first 2 years of the conferences from April 2017 to 2018 were conducted at KCL and the third conference was conducted in April 2019, at the University of East London (UEL). The fourth conference, which took place in September 2020, was our first online conference with Imperial College, London, because of the pandemic. We continued online for the 5th year, July 2021, the Open University co-hosted with the University of West London, and the sixth was our first hybrid (virtually and in-person) conference held at the University of West London, Ealing, and co-hosted with KCL.

## Impact from the first evaluations

It is important to highlight the impact of the conference and then demonstrate the evidence from delegates who completed the survey. The information is subsequently used to shape future conferences.

In brief, participants from the first conference were recently appointed lecturers, ECR, i.e., postdoctoral researchers, postgraduate students, and a few undergraduate students, and there were also non-academics happy to be in a space of BME researchers. All participants were either from London or outside London. Their experiences varied from either having a background in STEM or Arts and Humanities. Their motivations for attending were that they wanted to know who was in academia in terms of representation, if academia was for them and if so, what support was available to them. Many have become disillusioned with being in academia or considering academia as an option.

The first survey from the very first conference held in April 2017 consisted of 11 questions, the number of questions asked for future conferences was reduced, and this was to ensure that we got the salient information and also to encourage attendees to complete it as no one likes completing a long survey.

The first two questions sought to gain an idea of the audience and the university they came from. Attendees ranged from undergraduate students to non-academics. The universities that were part of the committee and the delegates that came after hearing about it came from University College London, Brunel University, Imperial College London, Kingston University, London School of Economics and Political Science, the Queen Mary University of London, the Royal Holloway University of London, Royal Veterinary College, the University of East London, Oxford Brookes, the University of Kent, the University of Essex, the University of Birmingham, Middlesex University, Brighton University, and the Goldsmiths University of London.

## Conference themes

People were asked to reflect on the following themes:

- Why they came to the conference?
- What tools they had before attending the conference, e.g., mentoring, networking, and applying for fellowships?
- What was their work–life balance like?
- Had the conference provided them with a better understanding of what it meant to remain in academia?

I will summarize the finding on each point and provide appropriate specific feedback from the delegate(s) to emphasize the point.

## Why they came to the conference?

People came to the conference because they wanted to hear and see inspirational speakers, and they wanted to learn tips and tools to remain in academia, whilst others said they wanted to network. “*I have been a postdoc for over 15 years and I found it difficult to move on to becoming an academic lecturer. The talk on how to get out of a slump and put bad experiences behind me has motivated me to try and get an academic position*.”

## What tools they had, e.g. mentoring, networking, and applying for fellowships?

The delegates were questioned about what tools they had before the conference, a high percentage, over 50% did not have a mentor nor applied for a fellowship but over 50% had been taking steps to build their network, “*the continuing emphasis on us being equipped to further ourselves is empowering and the mentoring focus was also very much appreciated*.”

## What was their work–life balance like?

Over 90% were mindful of their work–life balance, which has been attributed to their experiences in HE, “*if I need to be the best for my students and family, I need to look after my wellbeing*.”

## Had the conference provided them with a better understanding of what it meant to remain in academia?

Nearly 83% of the people agreed “*the honest practical, and down to earth attitude of all the range of speaker's whose stories were very different enough from each other, while reinforcing the conference's key messages.”*

## Impact from yearly evaluations

For the feedback over the subsequent 6 years, people were asked to reflect on the following themes:

- Did the overall organization of the conference meet your expectation?
- Has the conference provided you a better understanding of what you need to remain in academia?
- Has the conference increased your confidence and motivation to stay in academia?

**Figure 1:**
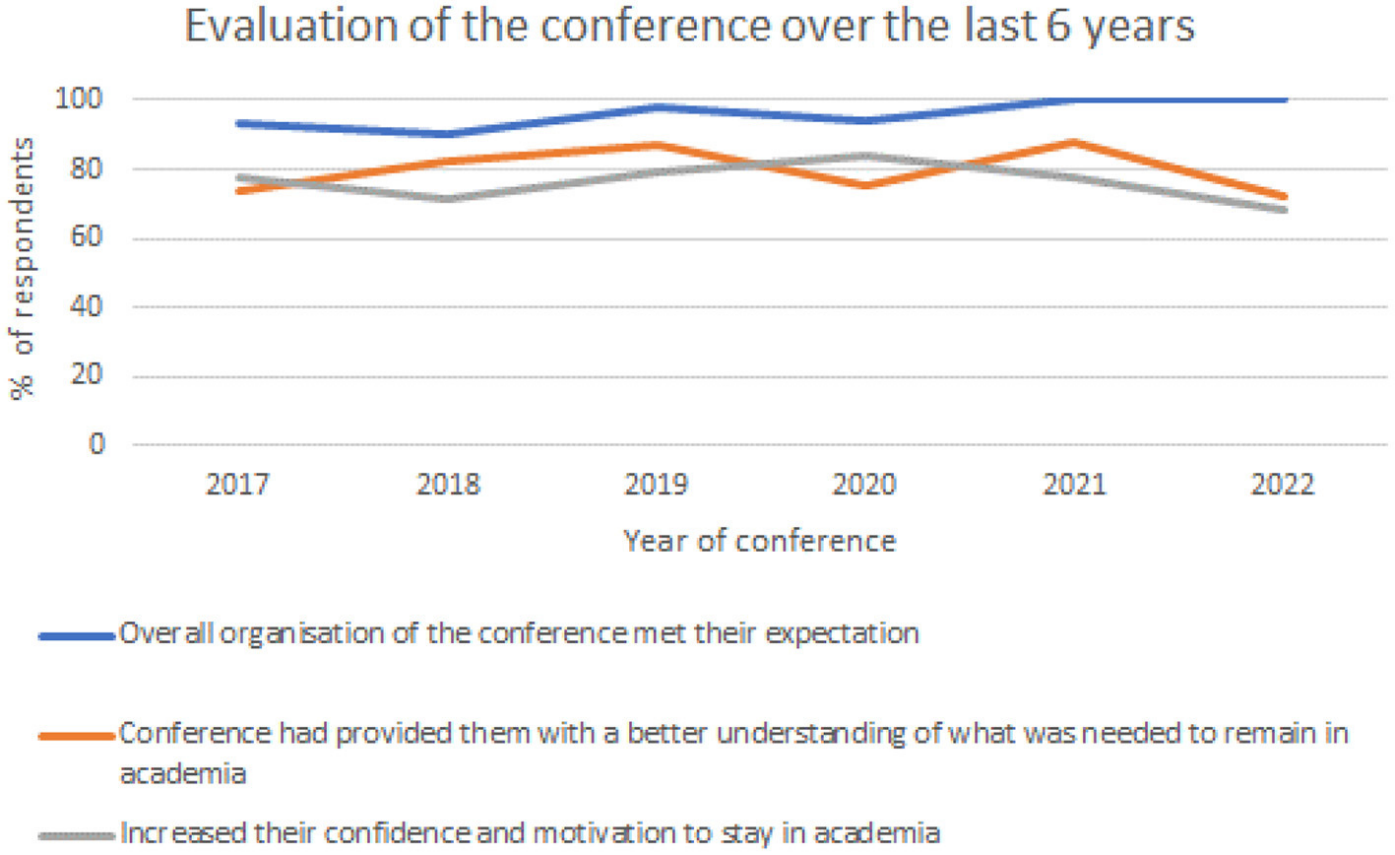
The data collected over the last 6 years and evaluates the conference using three questions.

I will summarize the following reflections over the years. The data are captured in Figure 1 which show the trends. The percentage feedback has been on average 40% over the 6 years.

## The power of mentorship and representation

In response to the first theme: ‘Did the overall organization of the conference meet your expectation?': the overall satisfaction has always been over 90%, but for the last 2 years, namely, 2021 and 2022, it was rated 100% which has been fantastic (Figure 1). When organizing these conferences, it is very hard to please every attendee, so this confirms the conference is indeed making a significant difference for every attendee.

The feedback over the years has been more positive than negative, “*It is a well organized event, and I would not change its concept or design,”* also “*to hear all the speakers talk so candidly and passionately about such personal experiences was an honor, so reassuring and an absolute privilege to hear,”* but some comments were made “*on providing advice that addressed older students concerns.”* This, therefore, demonstrates the power of mentorship and representation as mentioned earlier.

## Increasing the value of networks

There were comments on the focus of the conference being on STEM subjects, and some would have liked more of a balance, to incorporate social sciences/arts.

This is indeed a correct observation. The main sponsors for the conference have come from STEM backgrounds, and for the seventh conference, social science/arts organizations have been approached, to ensure it is more inclusive.

Comments were made on making the workshop sessions more interactive, and the facilitators of these sessions have been asked to address this, but my aim for these sessions was to ensure the delegates were empowered and know who to contact if more support was required. For example, feedback reported that “*It was quite interesting attending the conference. I am an ECR as this is my first teaching position. I had been a PostDoc for over 20 years prior to that. Even with this amount of experience in the industry, I was unaware of a lot of resources that are now available to BME researchers. This has been a great help for me.”*

“*I feel more inspired. Sense of belonging and community. Good to know that there are networks and support systems out there.”* This, therefore, demonstrates the importance of meeting new people, hence increasing your network, as your network is your net worth.

## The value of sharing lived experience

In response to “Has the conference provided you a better understanding of what you need to remain in academia,” Figure 1 shows the trends over the years which ranged from 72 to 88%. Each year brings different themes, speakers, and delegates, and anything over 70% suggests a high satisfaction which is great; however, I am always looking at the feedback on ways to improve.

The feedback included “*It opened my eyes to opportunities and obstacles in academia and how I need to navigate them*.”

“*I am not actually in academia but* have *considered going back for a long time. I felt disempowered and disengaged, but this conference has made me feel that I can leap when I am ready, and there is a network out there who will support me,”* which suggests that they have an understanding of what is needed to stay in academia, which includes the network, and mentors that can be obtained from the meeting people at the conference. This confirms by being in a space with people that have similar lived experiences you can challenge yourself to do what you thought originally was not possible.

## Shared resources—increasing confidence

In response to “Has the conference increased my confidence and motivation to stay in academia,” Figure 1 demonstrates that the responses range from 68% to 84%, and it fluctuates over the years, with 68% being the most recent conference and 84% back in 2020, whilst 2021 was 78%. Much has changed during the pandemic, and more conversations and possibly positive actions need to be taken.

I asked delegates to comment on the effectiveness of the hybrid conference, and the preferred attendance was in person. I note the majority of attendees for 2022 were online and that is possibly why the confidence and motivation to stay in academia dipped slightly as people have told me their preference is to attend in person. They said the experience is not the same as attending online, with specific emphasis made on networking. On the contrary, due to the cost of travel, as there were delegates that attended from the US and South Africa, their preference was to attend virtually.

Positive feedback includes the following:

“*This was my first and it felt like a very inviting and supportive space which was wonderful. I was inspired and engaged, and it boosted my desire to identify ways of mentoring students that want to come into research or academia (since I am an early career researcher so can't offer as much to colleagues yet).”*

“*Has encouraged me to stay in academia and advocate for relevant issues.”*

“*It has reinvigorated my desire to persevere and keep going in academia. I have been empowered and I'm reminded to use what I have because there are people who do a lot more with a whole lot less. Hugely really encouraging event! THANK YOU long may it continue!”*

“*It was beyond powerful seeing people who look like me in the same space working in academia. It's been so isolating in a way no one can ever understand unless you're from that particular background, so it was validating to have people who understood identified, and I could confide in. I also was very adamant on leaving academia, but this has made me realize it really is about the space you're in and working with people who look like you that get it!”*

“*I now feel I belong in academia knowing some of my international colleagues share the same dream with me.”*

To summarize, conference feedback showed increasing confidence amongst attendees through the many resources available within our community including obtaining a mentor, sponsor, and/or coach and increasing your network. Mentors will specifically share their journey and will provide cues to support you on your own. Having heard the story of how your mentor navigated their journey and the likely challenges they had to overcome, you will note perseverance is required. Moreover, understanding the bigger picture is empowering the future generations and being visible is powerful for BME students. The ethos of my conference is the three Ps, being positive, practical, and pragmatic. It will happen eventually, just do not give up.

## Second initiative—visibility of BME academics to senior promotion

The first initiative was to understand the barriers and challenges of BME postdoctoral researchers making the transition to become lecturers. On becoming a lecturer, the second initiative aimed to understand how do we make visible the BME academics and ensure that they are encouraged and supported to remain in academia. Secondly, they are promoted to senior academic positions, e.g., associate professor (reader) and professor.

I was appointed to participate in this initiative through a personal encounter with the former President of KCL, Sir Ed Byrne, with whom I spoke about my history at KCL. I mentioned not seeing an increase in the BME female staff teaching science, and through this face-to-face conversation, I was fortunate to have been given a temporary role at KCL within the Central Diversity and Inclusion team. My remit was to follow up on the KCL Race Equality Charter action plan which included designing methods to address the two initiatives.

I developed a method of engaging with 51 senior lecturers from various disciplines within KCL, who self-identified as BME. I started by sending an email introducing the project and asked whether they would like to participate in a telephone interview consisting of 13 questions, lasting for ~10 min. They were informed that the responses from the interview would form the basis for further questions at the focus group sessions. It would last 2 h for further exploration of the barriers and challenges facing BME senior lecturers in relation to their promotion to professors and possible solutions to address these.

## Interview topics

In brief, the topics in the interview referred to their experiences of applying for promotion, were they supported by their head of department, was the process transparent? were there obstacles? were they provided with a mentor? or did they feel valued?

Participant's roles were clinical and non-clinical. Fourteen were non-clinical, whereas six were clinical, three of which had mixed roles (clinical and non-clinical). The non-clinical role varied from faculties within KCL from health to arts and humanities.

To summarize the findings, many spoke about the importance of having a mentor—for personal development, but specifically for promotions. They wanted to have regular performance development and reviews.

The key themes included the following:

- support from the head of the department.
- transparency of promotions.
- obstacles to promotion.
- support for promotion.

## Support from the head of the department

The participants pointed out that support from the head of department (HoD) was limited, and many had observed a lack of noticeable attention/support from HoD compared to male and white staff. It was felt that there were cases where negative criticism was provided rather than positive criticism including encouragement and support. Some HoDs have been supportive and have been referred to as allies. If, however, more BME HoD were in place, this would dramatically impact staff seeking promotion as they would understand their challenges.

## Transparency of promotions

In terms of promotions, it was felt that promotions were not transparent, and the criteria were constantly changing including having a required number of PhD completions, a certain number of high-quality publications, and generating external funds. Such situations would result in limited promotions for BME staff (Morgan, 2016). They felt that the HoD should put in place initiatives to encourage BME staff to apply for promotion. They reported the lack of transparency in promotion, and the absence of feedback from the panel was apparent.

## Obstacles to promotion

Others felt that there were obstacles to promotion; BME staff felt hindered to apply for promotion at KCL. Some staff felt that they had a heavier than average teaching load, leaving no time for research and, therefore, a lack of grants and publications. Some felt overlooked for promotion compared to their white counterparts who had in some cases a lower number of grant/publication record/profile. It was felt that there was a variability in criteria within the same faculty. The same criteria should be in place for non-BME and BME staff, but too many times it varies; however, appreciation should be given to BME staff that engage in activities supporting diversity in the workplace as it is very common for BME staff to be involved in events.

## Support for promotion

Some female candidates felt that there should be leadership training for them, specifically designed for BME females, especially as it is known that some women lack confidence or suffer from imposter syndrome. Mentorship and leadership training should be used synonymously as it is extremely empowering to boost a woman's confidence.

Some BME staff did not feel as valued at KCL compared to experiences of institutions where they have previously worked, e.g., at Harvard/MIT, they were encouraged and supported throughout their career. Being valued enables you to enjoy your job and put a lot of effort into your daily activities, and that is how I feel in my current role. Students are inspired and and will impact on their career aspirations.

Personally, hearing the experiences of the senior lectures from the focus group conducted at KCL was hard; first, I did not think for a minute about the level at which they described their insecurities, discrimination, micro-aggressions, bullying, and victimization that were going on. I guess I have read about inequalities but never heard from senior academics at KCL. How was this to happen with policies in place to address it. I now understand why people for the sake of their well-being leave academia, but if we all leave who will be left to teach and inspire our young people struggling day in and day out. Leaving for me is just not an option. This focus group taught me what works and what does not.

I had no idea I would ever be in the position I am in now but was determined to support others through any initiatives I was involved with.

## Presenting recommendations

**Figure 2:**
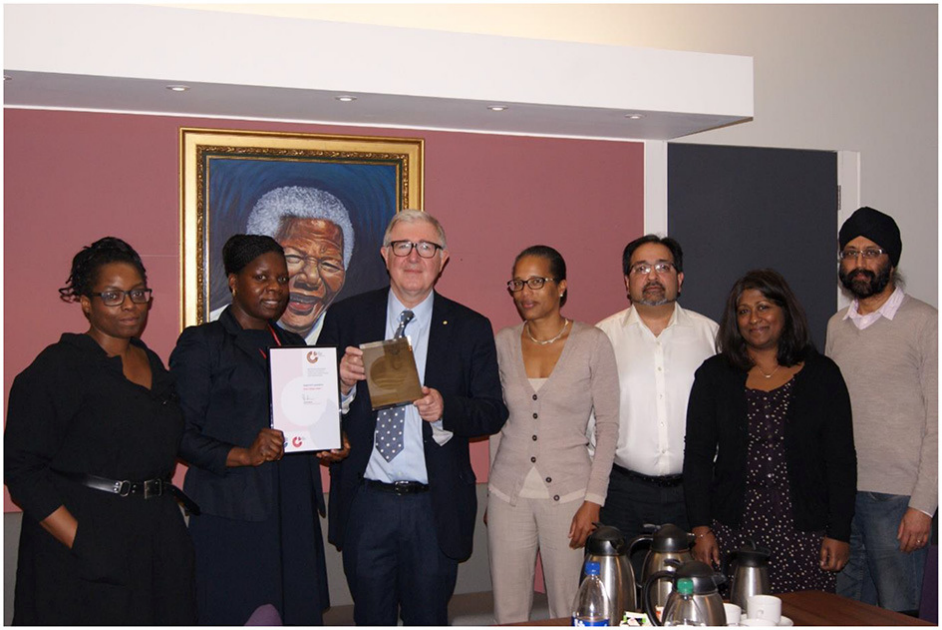
From L to R, Dr. Shubulade Smith, Dr. Bernadine Idowu, President and Principal Sir Ed Byrne, Professor Sylvane Desrivieres, Professor Kawal Rhode, Dr. Susan John, and Dr. Baljinder Mankoo (available at KCL on public internet page) (King's College London, 2016).

The findings and recommendations were presented to the then President of KCL Sir Ed Byrne, and he was happy to organize two informal meetings for those who had participated in the focus group. Two breakfast meetings were held in his office (Figure 2, one of our meetings).

He listened attentively to what the BME staff had to say, and I do recall him encouraging all the staff to either re-submit for promotion (those that had recently been rejected) or make the application for promotion, after hearing proof of their contribution to research, publications, grant awards. He also learnt of their contribution to diversity and inclusion in their various faculties or as part of a network.

After both meetings, I was curious to hear the thoughts of those who met the principal, and I asked them to provide feedback. I was pleased that they observed that he genuinely cared and wanted to ensure they are supported in their roles and of course are given the support to apply for promotion. Principals and senior leaders can indeed be influential in career progression, and it is called allyship (Macdonnell, 2020). My finding from the first initiative having had personal discussions with several delegates was with the support of senior leaders, and it supports them on their career progression.

## Outcome to date

From 2016 to date, there have been at least 10 promotions, one external to KCL, but most of the remaining at KCL are professors. The staff were extremely pleased to meet the principal and receive his words of support, and it encouraged them to proceed with the promotion. The staff are in turn supporting their peers through mentoring and reading their applications. In terms of shaping policy, I have been invited to numerous panels, Learning Teaching and Student Experience Conference by Chartered Association of Business schools (29.07.21) with people interested in learning more about how I can be influential in making a change in their field.

## Personal reflection

The title gives a personal reflection upon navigating into a senior academic role. Part of this discussion will include my experience on the back of the projects I got involved with. Listening to people's experience and having my mentors have guided me to my role. Let me explain. I was extremely fortunate to work part-time in diversity and inclusion at KCL after a chance meeting with Sir Ed Byrne, I made such an impact championing many projects related to race. They included the two groups I describe in this article, namely the BME ECR conference and Senior Lecturers focus group, but there were three other projects, including the Open doors project, which shone a spotlight on the contributions and achievements of BME staff and students, demonstrated through a dedicated website and taking photographs and placing on door panel around the various campuses, showing visibility.

Mentoring schemes for students and staff, where staff were matched with BME academics within universities, was part of the scheme. Lastly, refreshing the female Professor's frieze, they were all white women, but through a search within the university community, we were able to celebrate female professors from ethnic minorities backgrounds. I was living my best life, making an impact on things that I was extremely passionate about.

Figure 2 shows attendees of the focus group holding the Bronze Race Equality Charter Mark with the former Principal Sir Ed Byrne in his office.

Professor Marcia Wilson has contributed an excellent chapter in “Inside the Ivory Tower” edited by Wilson (1997). Her chapter “The search for that elusive sense of belonging, respect and visibility in academia” is so powerful. She started by sharing her own experience “*of searching for a feeling of belonging as a student and academic,”* and many years later BME students and staff are still feeling this (Idowu-Onibokun, 2021). In my current role at the University of West London, I was initially recruited as a consultant to develop a new school, with a new curriculum, a once in a lifetime opportunity. Too often BME students that choose to study a Biomedical Science degree give feedback on not seeing themselves reflected in the curriculum, the lecturers teaching them or relevance in the research field. This course offers all the above and more.

In December 2022, I attended a hybrid 2-day conference on “Decolonising and Diversifying Biosciences Education” at Cambridge organized by the Society for Experimental Biology (SEB). There I was able to share what decolonised and diversified courses looked like at UWL. I was asked what was needed to implement that and I explained the importance of having supportive allies. The Provost and Senior Deputy Vice-Chancellor, Professor Anthony Woodman, is an example of an ally at UWL, and he recruited consultants with the understanding that more diversity needs are to be fulfilled to address the challenges and inequalities within academia. After 2 years, we can boast of a diverse curriculum, diverse staff body, diverse research interest, and a diverse student body. I can confidently and proudly say our School of Biomedical Sciences at the University of West London is a model department. At KCL, the previous President and Principal Sir Ed Byrne was another person who was an ally. He listened to me when I spoke and gave me a voice.

I have been invited on several occasions to sit on panels discussing best practices from our school in learning and teaching, decolonising the curriculum and even recorded podcasts, for example, on the experience of Black researchers in Science and Technology for the Science and Foundation Technology (SFT).

To address and make changes, we need allies as examples mentioned above and also mentors. I have several mentors, and I wish to make mention of those advising me on the conference such as Professor Funmi Olonisakin (VP Global, KCL) and Professor Marcia Wilson (Dean, Open University) who have been on this journey with me from the beginning. Professor Wilson sponsored the conference when working at the University of East London in April 2019 and at the Open University in September 2021. These visionaries are just amazing. It was Dr. Donald Palmer (Associate Professor, RVC), who prophesied in 2017 after the first conference, “*you have started a revolution, you cannot stop now!*”

Finally, I would like to end on a personal note if I may, I am fully aware of the injustices in this world, the discriminations, racism and the list is endless, but on the other hand, being a woman of faith has given me a focus. When God made me, He knew why, I believe what I am doing is what I have been ordained to, it is my vocation, my calling.

## Conclusion

Universities, similar to UWL, should put initiatives in place to make sure all students feel they are supported that they have a sense of belonging. Peer mentoring should be available for all students, and this will assist BME students to obtain the top grades required to go onto postgraduate study. On obtaining their PhD, they should be guided to consider an academic career to increase the pool of professors of both genders. Currently, there are only 40 Black female professors out of 6,340. The BME ECR conference is empowering people to use the tools, for example, mentoring and networking to remain and succeed in academia. One of the attendees gave the following feedback: “*the conference has been found to be not patronizing, divisive, or tokenistic, but the best professional development event I have been to*.”

## Future

The pandemic has had negative and positive outcomes. One of the positives is my conference has officially gone global. I have had interests in South Africa, America, and Australia. Next year, for the first time in 6 years, we shall be going outside London, which was always the plan; our 7th BME ECR will be co-hosted by the University of Leeds and the University of West London, annually supported by the Center for Doctoral studies and Center for Research Staff Development at King's College, London.

## Data availability statement

The raw data supporting the conclusions of this article will be made available by the authors, without undue reservation.

## Ethics statement

Ethical review and approval was not required for the study involving human participants in accordance with the local legislation and institutional requirements. Written informed consent to participate in this study was not required from the participants in accordance with the national legislation and the institutional requirements.

## Author contributions

The author confirms being the sole contributor of this work and has approved it for publication.
